# Timing of Bronchoscopy and Plasma Microbial Cell-Free DNA Sequencing in Immunocompromised Host Pneumonia

**DOI:** 10.1093/ofid/ofag361

**Published:** 2026-06-18

**Authors:** Ahmad Mourad, Daniel S Lupu, Morgan Richey, Paul Steven, Vance G Fowler, Brad Perkins, Thomas L Holland, Stephen P Bergin

**Affiliations:** Division of Infectious Diseases, Department of Medicine, Duke University School of Medicine, Durham, North Carolina, USA; Duke Clinical Research Institute, Durham, North Carolina, USA; Karius Inc, Redwood City, California, USA; Karius Inc, Redwood City, California, USA; North American Science Associates Ltd, Selby, United Kingdom; Division of Infectious Diseases, Department of Medicine, Duke University School of Medicine, Durham, North Carolina, USA; Duke Clinical Research Institute, Durham, North Carolina, USA; Karius Inc, Redwood City, California, USA; Division of Infectious Diseases, Department of Medicine, Duke University School of Medicine, Durham, North Carolina, USA; Duke Clinical Research Institute, Durham, North Carolina, USA; Duke Clinical Research Institute, Durham, North Carolina, USA; Division of Pulmonary, Allergy, and Critical Care Medicine, Department of Medicine, Duke University School of Medicine, Durham, North Carolina, USA

**Keywords:** bronchoscopy, diagnostics, immunocompromised pneumonia, microbial cell-free DNA metagenomic sequencing

## Abstract

**Background:**

Immunocompromised patients are at high risk of pneumonia, with associated poor outcomes. Rapid microbiologic diagnosis is crucial, yet diagnostic yields vary widely. We evaluated the variability in diagnostic yield of usual care testing and plasma microbial cell-free DNA (mcfDNA) sequencing in the prospective observational Pneumonia in the Immunocompromised—Use of the Karius Test for the Detection of Undiagnosed Pathogens (PICKUP) study, specifically focusing on timing of testing relative to the onset of pneumonia.

**Methods:**

In this exploratory analysis, patient characteristics, variability in diagnostic yield, and the timing of bronchoscopy and mcfDNA sequencing from date of first abnormal imaging associated with suspected pneumonia were evaluated across enrolling sites.

**Results:**

A total of 222 patients from 10 enrolling sites were analyzed. Usual care diagnostic yield varied across sites (range, 7.7%–57.7%). Patient characteristics did not differ between sites, and median time from abnormal imaging to bronchoscopy was not different across sites (3 days [IQR, 3]). Diagnostic yield of bronchoscopy was significantly higher when performed ≤3 days (early) from abnormal imaging (38.5% [52/135]) versus >3 days (delayed) (21.8% [19/87]) (difference, 16.7% [95% CI, 2.5%–28.3%]; *P* = .009). Adding mcfDNA sequencing to usual care testing increased overall diagnostic yield by 7.9% for patients undergoing early bronchoscopy, and by 16.3% for delayed bronchoscopy.

**Conclusions:**

Early bronchoscopy enhances diagnostic yield in immunocompromised patients with suspected pneumonia. Irrespective of timing, plasma mcfDNA sequencing increases overall diagnostic yield in this clinical scenario. These findings underscore the importance of prompt diagnostic strategies in this patient population.

Pneumonia is a common cause of infection-related morbidity and mortality in patients with hematologic malignancies, resulting in a 1-year mortality of up to 80% in those requiring intensive care [1]. Delayed microbiological diagnosis can be associated with poor outcomes in these immunocompromised patients [1, 2]. The current landscape of diagnostic testing in this vulnerable population lacks comprehensive and effective options [3]. Clinicians often pursue invasive diagnostic procedures—that is, bronchoscopy with bronchoalveolar lavage (BAL)—to improve microbiologic diagnostic yield [4–8]. Current data suggest that early bronchoscopy, within 5 days from the identification of pulmonary complications in immunocompromised patients, significantly improves diagnostic yield [9]. However, even early bronchoscopy may fail to identify a cause of pneumonia [9], and rates of complications from bronchoscopy in this population can be high [10].

Microbial cell-free DNA (mcfDNA) sequencing has emerged as a noninvasive microbiological diagnostic testing option for immunocompromised patients [11–14] and patients with pneumonia [15]. The prospective, multicenter, observational Pneumonia in the Immunocompromised—Use of the Karius Test for the Detection of Undiagnosed Pathogens (PICKUP) study evaluated whether plasma mcfDNA sequencing improved diagnostic yield in patients with hematologic malignancies or hematopoietic cell transplantation (HCT) undergoing bronchoscopy in an attempt to establish a microbial etiology of clinically suspected pneumonia [14]. In PICKUP, usual care testing identified a cause of pneumonia in 30% (52/173) of patients included in the per-protocol analysis. Plasma mcfDNA identified a cause of pneumonia in 28% (49/173) of included patients, with mcfDNA exclusively identifying an etiology of pneumonia in 21 of 173 patients—increasing the overall diagnostic yield by 12% (95% confidence interval [CI], 7.7%–18.0%; *P* < .001). Importantly, however, significant variation in both usual care and plasma mcfDNA diagnostic yield was observed across enrolling sites.

Utilizing data from the PICKUP study, we conducted a post hoc exploratory analysis to identify drivers of diagnostic yield variability, specifically focusing on whether timing of bronchoscopy from first identified abnormal imaging associated with clinically suspected pneumonia impacted the diagnostic yield of usual care testing. Additionally, we evaluated whether the timing of specimen collection for plasma mcfDNA sequencing impacted diagnostic yield.

## METHODS

### Ethical Statement

The PICKUP study protocol was approved by the institutional review boards at each enrolling site.

### Study Design and Definitions

The design, rationale, and protocol of the PICKUP study (NCT04047719) have been published [14]. In brief, the PICKUP study was a prospective, multicenter, observational study conducted at 10 tertiary care centers in the United States. The primary objective of the study was to determine the additive diagnostic value of plasma mcfDNA in immunocompromised patients undergoing bronchoscopy for the microbiological diagnosis of suspected pneumonia—defined as the proportion of patients with an etiology of pneumonia exclusively identified by plasma mcfDNA sequencing. Hospitalized patients who were receiving treatment for hematological malignancies, those who recently underwent HCT, or those receiving immunosuppressive therapy for graft-versus-host disease were eligible for enrollment. A protocol-specified minimum diagnostic standard panel of tests was defined for all patients, which included blood culture, serum galactomannan, multiplex polymerase chain reaction panel for respiratory viruses, BAL *Pneumocystis jirovecii* testing, BAL gram stain and bacterial culture, BAL fungal stain and culture, and BAL acid-fast bacilli smear and culture [14]. Usual care, defined as the protocol-specified minimum diagnostic standard and any additional diagnostic testing collected as part of the patient's clinical care, was divided into noninvasive and invasive (ie, tests performed on specimens obtained by bronchoscopy, herein referred to as “bronchoscopy”) testing. Plasma mcfDNA sequencing test was collected within 24 hours of enrollment. A centralized committee of infectious diseases and pulmonary physicians adjudicated the probable microbiologic cause of pneumonia in a 2-step process; first with the results of usual care testing alone, and second with the results of plasma mcfDNA metagenomic sequencing.

For this study, we conducted a post hoc exploratory analysis of patients enrolled in the PICKUP study, for whom clinical adjudication of the cause of pneumonia was completed, to identify factors that may have impacted the diagnostic yield (ie, proportion of patients with an identified pathogen adjudicated as the probable cause of pneumonia) of bronchoscopy and plasma mcfDNA sequencing—with a focus on timing of bronchoscopy from the first abnormal imaging associated with the episode of pneumonia during the enrollment clinical encounter. Given that patients who had a cause of pneumonia identified with noninvasive diagnostic testing prior to bronchoscopy were excluded from PICKUP, we did not evaluate noninvasive usual care diagnostic testing in this study. Record review was performed (A.M.) to determine the date of abnormal imaging associated with clinically suspected pneumonia during the encounter.

### Plasma Microbial Cell-Free DNA Sequencing

The Karius test has been validated to both detect and quantify mcfDNA in plasma [16]. Clinicians caring for the patients enrolled in the PICKUP study were blinded to the results of plasma mcfDNA sequencing. As such, plasma mcfDNA sequencing did not impact the clinical care of these patients.

### Variables and Outcome Measures

The date of first abnormal imaging associated with clinically suspected pneumonia during the enrollment encounter was defined as the first day that any chest X-ray or computed tomography scan with findings consistent with viral, bacterial, or fungal pneumonia was obtained [17]. These findings included, but were not limited to, lobar or multifocal infiltrates, consolidations, ground-glass opacities, nodules, cavitary lesions, or pleural effusions. Days from the onset of abnormal imaging to bronchoscopy and specimen collection were calculated. Diagnostic yield for bronchoscopy and plasma mcfDNA sequencing was calculated as a proportion of enrolled patients who had an adjudicated probable cause of pneumonia identified by the corresponding testing.

### Statistical Analysis

Statistical analysis was performed using SAS version 9.4 (SAS Institute). Descriptive statistics were calculated using mean, median, and interquartile ranges for continuous variables. Comparisons were performed by calculating *P* values and confidence intervals (CIs) via the Score (Farrington–Manning) method to assess associations between diagnostic yield and test timing.

## RESULTS

**Figure 1.**
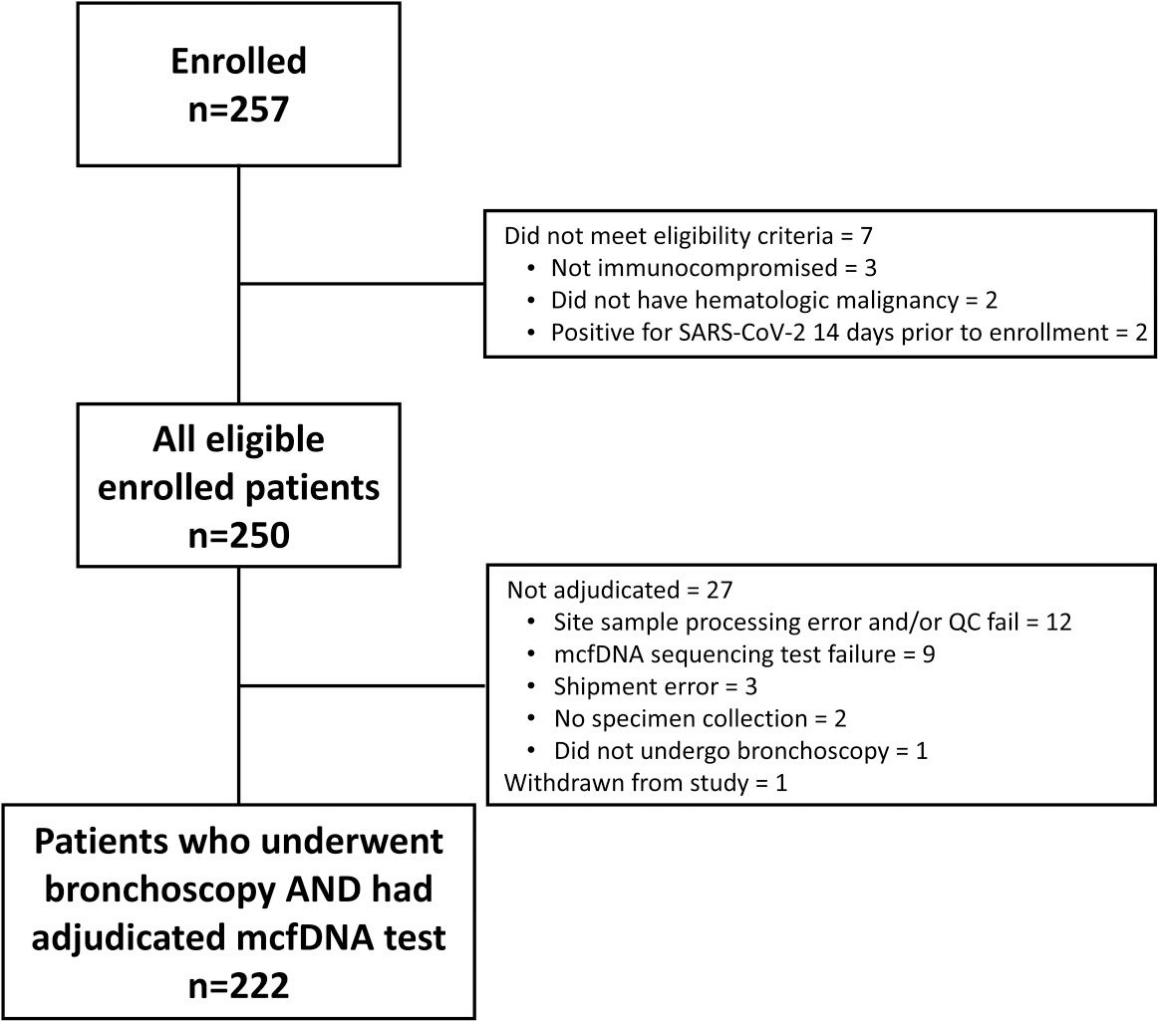
Consolidated Standards of Reporting Trials (CONSORT) diagram of eligible patients. Abbreviations: mcfDNA, plasma microbial cell-free DNA; QC, quality control; SARS-CoV-2, severe acute respiratory syndrome coronavirus 2.

Of the 257 patients enrolled in PICKUP, 222 were eligible for this exploratory analysis (Figure 1). Key patient characteristics including type of hematologic malignancy, HCT status, prior chemotherapy received, and prior antimicrobial exposure were similar across enrolling sites (Table 1).

**Table 1.** Patient Characteristics

Characteristic	Site A (n = 8)	Site B (n = 75)	Site C (n = 15)	Site D (n = 11)	Site E (n = 26)	Site F (n = 5)	Site G (n = 22)	Site H (n = 38)	Site I (n = 9)	Site J (n = 13)	Total (N = 222)
Age, y, median (IQR)	61.5 (13.5)	63.0 (20.0)	61.0 (12.0)	61.0 (20.0)	65.0 (10.0)	59.0 (27.0)	56.5 (22.0)	60.5 (24.0)	68.0 (11.0)	52.0 (27.0)	62.0 (19.0)
Sex											
Female	3 (37.5)	18 (24.0)	5 (33.3)	6 (54.5)	9 (34.6)	1 (20.0)	8 (36.4)	14 (36.8)	3 (33.3)	5 (38.5)	72 (32.4)
Male	5 (62.5)	57 (76.0)	10 (66.7)	5 (45.5)	17 (65.4)	4 (80.0)	14 (63.6)	24 (63.2)	6 (66.7)	8 (61.5)	150 (67.6)
Hematologic malignancy type											
Leukemia	3 (37.5)	57 (76.0)	9 (60.0)	7 (63.6)	17 (65.4)	4 (80.0)	12 (54.5)	28 (73.7)	5 (55.6)	9 (69.2)	151 (68.0)
Lymphoma	3 (37.5)	21 (28.0)	3 (20.0)	2 (18.2)	2 (7.7)	3 (60.0)	4 (18.2)	7 (18.4)	2 (22.2)	0 (0)	47 (21.2)
Multiple myeloma	0 (0)	4 (5.3)	3 (20.0)	1 (9.1)	3 (11.5)	0 (0)	5 (22.7)	3 (7.9)	1 (11.1)	1 (7.7)	21 (9.5)
Myelodysplastic syndrome	5 (62.5)	15 (20.0)	2 (13.3)	3 (27.3)	7 (26.9)	0 (0)	0 (0)	4 (10.5)	1 (11.1)	3 (23.1)	40 (18.0)
Prior hematopoietic cell transplant											
Allogeneic	3 (37.5)	14 (18.7)	6 (40.0)	3 (27.3)	6 (23.1)	1 (20.0)	8 (36.4)	12 (31.6)	2 (22.2)	4 (30.8)	59 (26.6)
Autologous	0 (0)	1 (1.3)	0 (0)	1 (9.1)	2 (7.7)	0 (0)	4 (18.2)	1 (2.6)	0 (0)	0 (0)	9 (4.1)
Relapsed hematologic malignancy at enrollment	7 (87.5)	54 (72.0)	9 (60.0)	4 (36.4)	8 (30.8)	1 (20.0)	14 (63.6)	26 (68.4)	1 (11.1)	4 (30.8)	128 (57.7)
Chemotherapy received within 45 d of enrollment	6 (75.0)	67 (89.3)	13 (86.7)	7 (63.6)	24 (92.3)	3 (60.0)	12 (54.5)	24 (63.2)	8 (88.9)	7 (53.8)	171 (77.0)
Prior^a^ anti-infective therapy at enrollment											
Anti-pseudomonal antibacterial	8 (100)	74 (98.7)	14 (93.3)	10 (90.9)	25 (96.2)	5 (100)	22 (100)	36 (94.7)	8 (88.9)	13 (100)	215 (96.8)
Anti-MRSA antibacterial	7 (87.5)	68 (90.7)	13 (86.7)	10 (90.9)	21 (80.8)	5 (100)	14 (63.6)	25 (65.8)	4 (44.4)	12 (92.3)	179 (80.6)
Mold-active antifungal	7 (87.5)	63 (84.0)	12 (80.0)	10 (90.9)	22 (84.6)	2 (40.0)	11 (50.0)	21 (55.3)	8 (88.9)	12 (92.3)	168 (75.7)
*Pneumocystis jirovecii* prophylaxis	0 (0)	10 (13.3)	2 (13.3)	1 (9.1)	0 (0)	1 (20.0)	2 (9.1)	4 (10.5)	0 (0)	5 (38.5)	25 (11.3)
Days to bronchoscopy from abnormal imaging, median (IQR)	5.0 (8.0)	3.0 (3.0)	2.0 (2.0)	4.0 (9.0)	2.0 (3.0)	2.0 (3.0)	3.5 (4.0)	2.0 (2.0)	4.0 (2.0)	3.0 (2.0)	3.0 (3.0)
30-d mortality	1 (12.5)	7 (9.3)	7 (46.7)	0 (0)	4 (15.4)	1 (20.0)	2 (9.1)	10 (26.3)	2 (22.2)	2 (15.4)	36 (16.2)

Data are presented as No. (column %) unless otherwise indicated.

Abbreviations: IQR, interquartile range; MRSA, methicillin-resistant *Staphylococcus aureus*.

^a^In the 7 days prior to enrollment.

**Figure 2.**
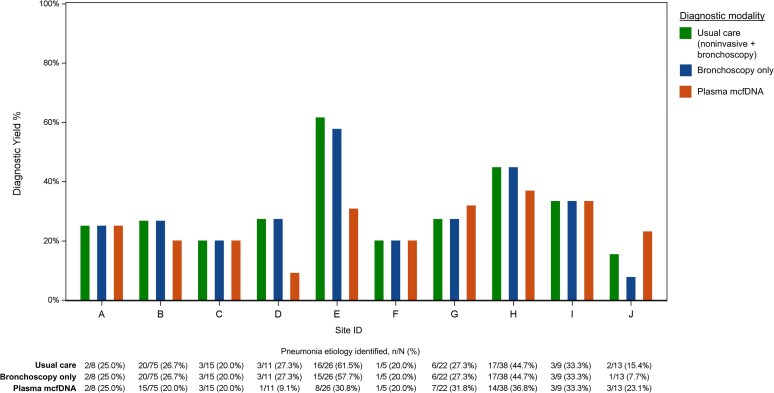
Diagnostic yield of usual care (noninvasive diagnostic testing + bronchoscopy), bronchoscopy only, and plasma microbial cell-free DNA (mcfDNA) sequencing by enrollment site.

**Figure 3.**
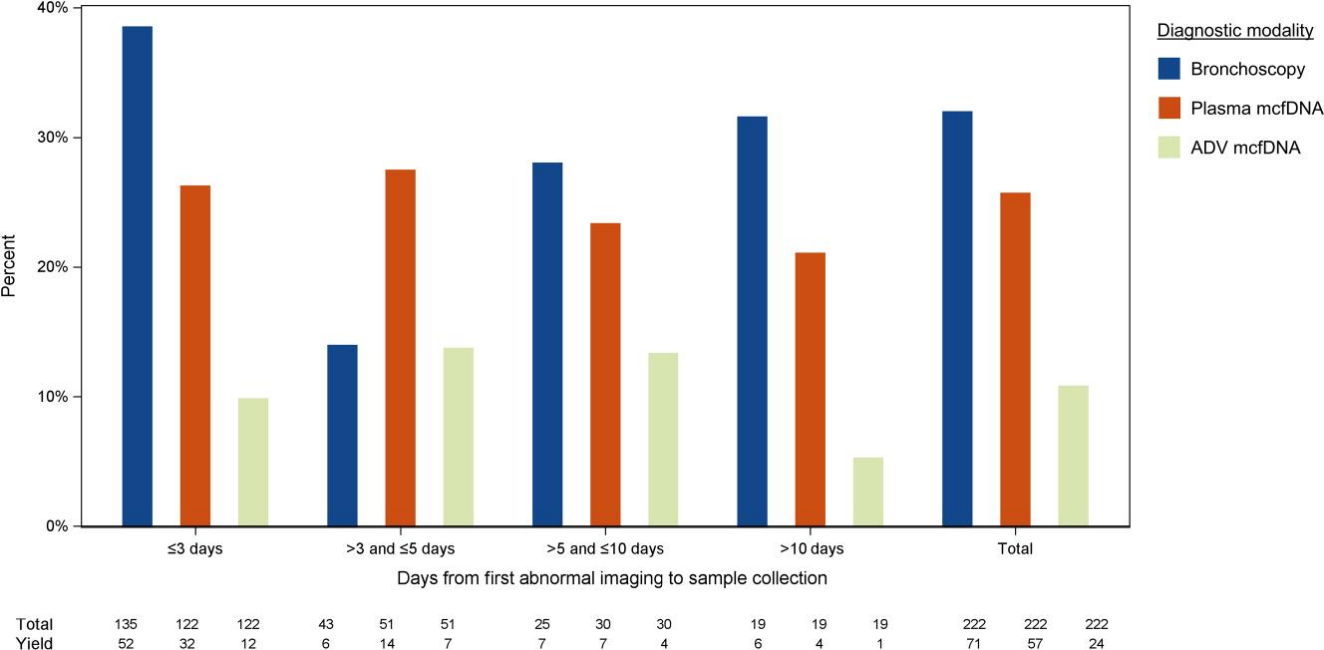
Diagnostic yield of bronchoscopy, plasma mcfDNA sequencing, and additive diagnostic value (ADV) of plasma microbial cell-free DNA (mcfDNA) by time from first abnormal imaging to bronchoscopy and plasma specimen collection.

Overall, the diagnostic yield of bronchoscopy was 32% (71/222), and plasma mcfDNA sequencing 25.7% (57/222). There was variability both in the diagnostic yield of bronchoscopy (range, 7.7%–57.7%), and plasma mcfDNA sequencing (range, 9.1%–36.8%) across the 10 enrollment sites (Figure 2). Plasma mcfDNA sequencing exclusively identified an adjudicated probable cause of pneumonia in 24 of 222 (10.8%) patients (Figure 3). A detailed list of adjudicated causes of pneumonia and identified microbes is included in the supplement (Supplementary Table 2).

The overall median time from first abnormal imaging to bronchoscopy was 3 (IQR, 3) days and was not different across the enrolling sites, with 80% (178/222) of patients undergoing bronchoscopy ≤5 days from abnormal imaging (Table 1). Diagnostic yield of bronchoscopy was not significantly different when performed ≤5 days from abnormal imaging (32.6% [58/178]) versus >5 days (29.6% [13/44]) (difference, 3.0% [95% CI, −13.8% to 17.4%]; *P* = .699) (Supplementary Table 1). However, diagnostic yield of bronchoscopy was significantly improved when performed ≤3 days from abnormal imaging (38.5% [52/135]) versus >3 days (21.8% [19/87]) (difference, 16.7% [95% CI, 2.5%–28.3%]; *P* = .009). Diagnostic yield of plasma mcfDNA remained consistent when the plasma specimen was collected ≤5 days from abnormal imaging (26.6% [46/173]) versus >5 days (22.4% [11/49]) (difference, 4.2% [95% CI, −10.6% to 16.0%]; *P* = .56), or ≤3 days from abnormal imaging (26.2% [32/122]) versus >3 days (25% [25/100]) (difference, 1.2% [95% CI, −10.3% to 12.8%]; *P* = .83) (Figure 3). Furthermore, the additive diagnostic value of plasma mcfDNA was similar when the plasma specimen was collected ≤3 days from abnormal imaging (9.8% [12/122]) versus >3 days (12% [12/100]) (difference, 2.2% [95% CI, −6.1% to 10.4%]; *P* = .61) (Figure 3).

For plasma mcfDNA, distribution of pathogen categories (viral, bacterial, and fungal) identified as the probable cause of pneumonia varied according to time from first abnormal imaging to specimen collection. In earlier time periods, viral pathogens comprised a greater proportion of detected etiologies compared to later time periods, whereas the relative contribution of bacterial and fungal pathogens increased with longer intervals to testing (Supplementary Figure 1).

Of note, the overall positive percent agreement (52% [27/52], 95% CI, 38%–66%) and negative percent agreement (83% [100/121], 95% CI, 75%–89%) between bronchoscopy and plasma mcfDNA in detecting the adjudicated probable cause of pneumonia in the per-protocol population of the PICKUP study has been previously reported in detail, including by pathogen category [14].

## DISCUSSION

In this exploratory analysis of the PICKUP study conducted in immunocompromised patients, specifically those with hematologic malignancies or HCT, with suspected pneumonia, we identified 3 key findings: (1) although all patients were enrolled from tertiary medical centers and had similar characteristics, the diagnostic yield of usual care testing, including bronchoscopy, varied substantially between enrolling sites; (2) early bronchoscopy, within 3 days of abnormal imaging associated with clinically suspected pneumonia, was associated with improved microbiologic diagnostic yield; and (3) plasma mcfDNA sequencing improved diagnostic yield both early and later in the course of pneumonia. These findings suggest that a strategy of combining early bronchoscopy with plasma mcfDNA sequencing can improve diagnostic yield in immunocompromised patients with suspected pneumonia, potentially improving their clinical care.

The findings of this analysis provide important insights into the diagnostic yield of bronchoscopy in immunocompromised patients with suspected pneumonia as it relates to the timing of diagnostic procedures. While prior literature has suggested that bronchoscopy within 5 days improves diagnostic yield [9], our results suggest that bronchoscopy conducted sooner—within 3 days of abnormal imaging—significantly improves the likelihood of identifying a probable microbiologic cause of pneumonia, and that the yield was not improved beyond that relatively short window. The discrepancy between these results may reflect the dynamic nature of pulmonary infections in immunocompromised patients, which could be impacted by factors such as empiric antimicrobial treatment. Importantly, however, given that 80% of patients in our study underwent bronchoscopy within 5 days from abnormal imaging, we may have been underpowered to detect a difference at later time points. Nevertheless, these findings have potential clinical implications for the timing of diagnostic procedures in immunocompromised patients, where a rapid and accurate diagnosis is essential for guiding appropriate treatment.

In addition to bronchoscopy, plasma mcfDNA sequencing contributed an overall additive diagnostic value of approximately 11%. While the yield of plasma mcfDNA sequencing was higher when performed within 3 days (26.2% [32/122]), compared to >10 days (21.1% [4/19]) from abnormal imaging associated with pneumonia, plasma mcfDNA can be a useful noninvasive testing option both early and later in the course of pneumonia. This highlights the potential of plasma mcfDNA as a complementary diagnostic tool, especially in cases where bronchoscopy is either contraindicated, delayed, or nondiagnostic. Additionally, preliminary results of mcfDNA sequencing on BAL specimens have also shown early promise in identifying causative pathogens and may be a future strategy to employ in patients undergoing bronchoscopy [18].

**Figure 4.**
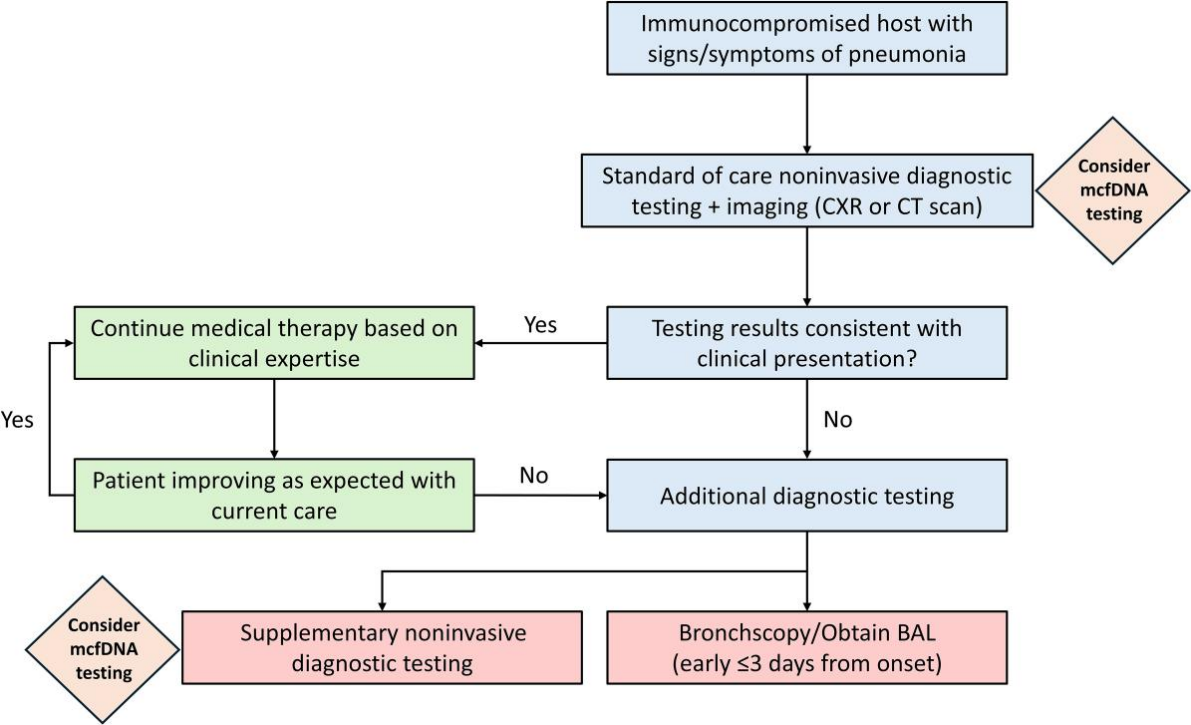
Proposed diagnostic approach to timing of bronchoscopy and plasma mcfDNA sequencing in immunocompromised patients with suspected pneumonia. Abbreviations: BAL, bronchoalveolar lavage; CT, computed tomography; CXR, chest X-ray; mcfDNA, plasma microbial cell-free DNA.

Incorporating our results into the framework for diagnosis of immunocompromised host pneumonia put forth by Cheng and colleagues [17], we propose that bronchoscopy should be performed within 3 days of onset of suspected pneumonia when possible, and that plasma mcfDNA can be used as both an early and/or late diagnostic tool within this algorithm (Figure 4). Additionally, plasma mcfDNA should be considered when bronchoscopy may not be feasible.

### Limitations

Our study has several limitations. First, PICKUP only included patients with hematologic malignancies or HCT. As such, these findings may not be applicable to other immunocompromised patient populations. Second, the date of first abnormal imaging associated with clinically suspected pneumonia was limited to radiographic images obtained during the clinical encounter in which patients were enrolled in the PICKUP study. This could have excluded abnormal images associated with suspected pneumonia that were obtained prior to this clinical encounter and therefore provided an underestimate of the time from first abnormal imaging to bronchoscopy. Third, given that empiric antimicrobial therapy was nearly universal among this patient population, we could not evaluate the impact of empiric antimicrobials on diagnostic yield of either bronchoscopy or plasma mcfDNA over time. Of note, the impact of prior anti-infective therapy on the yield of plasma mcfDNA sequencing in this specific context is unknown. However, other studies, including those in patients with bloodstream infections, have shown that the yield and clinical impact of plasma mcfDNA sequencing decreased following initiation of anti-infective therapy [19–21]. Additionally, because patients with a potential cause of pneumonia identified by preliminary noninvasive testing were excluded, this study may underestimate the diagnostic yield of noninvasive testing, making it challenging to draw conclusions about the utility of these tests in this population. Furthermore, we were not able to explore the extent of diagnostic testing beyond the protocol-specified minimum diagnostic standard as a potential driver of diagnostic yield variability across enrolling sites. Finally, given the study design, we could not assess the clinical impact of earlier microbiologic diagnosis as it pertains to appropriate antimicrobial therapy or patient-centered clinical outcomes.

## CONCLUSIONS

Overall, our study underscores the importance of early bronchoscopy in enhancing diagnostic yield in immunocompromised patients with suspected pneumonia and highlights the value of plasma mcfDNA sequencing as a tool that can enhance management both early and late in the pneumonia course. These findings contribute to the growing body of evidence supporting early and comprehensive diagnostic strategies in high-risk patients, with the potential to improve clinical outcomes in this vulnerable population.

## Supplementary Material

ofag361_Supplementary_Data

## Supplementary Data

Supplementary materials are available at *Open Forum Infectious Diseases* online. Consisting of data provided by the authors to benefit the reader, the posted materials are not copyedited and are the sole responsibility of the authors, so questions or comments should be addressed to the corresponding author.
