# Supplementary material for: Timing of Bronchoscopy and Plasma Microbial Cell-Free DNA Sequencing in Immunocompromised Host Pneumonia
Source: Open Forum Infect Dis. 2026 Jun 18;13(6):ofag361. doi: 10.1093/ofid/ofag361 (PMC13276870; doi:10.1093/ofid/ofag361)
Supplement: ofag361_Supplementary_Data [file ofag361_supplementary_data.docx]

**Supplementary Appendix**

Supplemental Table 1……………………………………………………………………………………………………………………………………………….Page 2

Supplemental Figure 1……………………………………………………………………………………………………………………………………………..Page 3

Supplemental Table 2……………………………………………………………………………………………………………………………………………….Page 4

**Supplemental Table 1.** Difference in diagnostic yield of bronchoscopy by timing from first abnormal imaging.

| **Diagnostic yield of bronchoscopy by number of days from abnormal imaging** | | **Difference in diagnostic yield** | **95% CI** | **p-value** |
| --- | --- | --- | --- | --- |
| **≤1 days** | **>1 days** |  |  |  |
| 45.2% (19/42) | 28.9% (52/180) | 16.4% | -0.8% – 33.4% | 0.041 |
|  |  |  |  |  |
| **≤2 days** | **>2 days** |  |  |  |
| 41.6% (42/101) | 24.0% (29/121) | 17.6% | 3.4% – 30.0% | 0.005 |
|  |  |  |  |  |
| **≤3 days** | **>3 days** |  |  |  |
| 38.5% (52/135) | 21.8% (19/87) | 16.7% | 2.5% – 28.3% | 0.009 |
|  |  |  |  |  |
| **≤4 days** | **>4 days** |  |  |  |
| 35.7% (56/157) | 23.1% (15/65) | 12.6% | -1.7% – 24.8% | 0.067 |
|  |  |  |  |  |
| **≤5 days** | **>5 days** |  |  |  |
| 32.6% (58/178) | 29.6% (13/44) | 3.0% | -13.8% – 17.4% | 0.699 |
|  |  |  |  |  |

**Supplemental Figure 1.** Differences in diagnostic yield by pathogen type for invasive usual care testing, plasma mcfDNA sequencing, and additive diagnostic value (ADV) of plasma mcfDNA by time from first abnormal imaging to specimen collection.

**
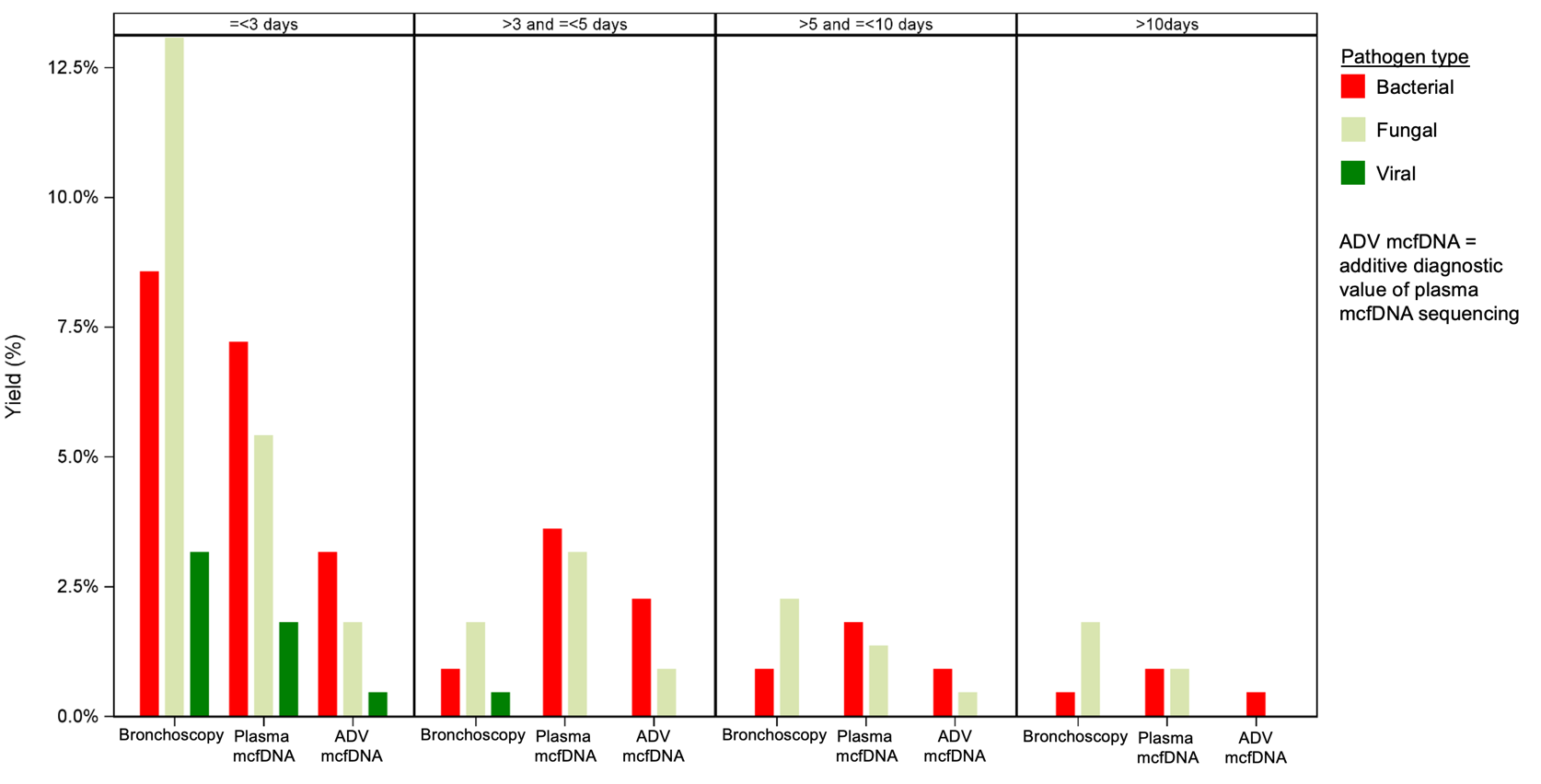
**

**Supplemental Table 2.** Adjudicated causes of pneumonia by usual care and by plasma mcfDNA sequencing.

- *Microbe names in green text were adjudicated as a probable cause of pneumonia;* ***bold text*** *represents agreement between Usual Care and plasma mcfDNA sequencing test results.*
- *Microbe names in blue text were adjudicated as a clinically relevant infection other than pneumonia.*
- *Microbe names in red text were adjudicated as not causing an active infection (commensal organism or contaminant).*

|  | **Adjudicated Cause of Pneumonia** | |
| --- | --- | --- |
| **Patient** | **By Usual Care** | **By mcfDNA** |
| 1 | ***Mycobacterium avium/intracellulare*** | ***Mycobacterium avium/intracellulare***  BK Polyomavirus  Human Herpesvirus 6 |
| 2 | ***Staphylococcus aureus*** | ***Staphylococcus aureus***  CMV |
| 3 | ***E. coli*** | ***E. coli*** |
| 4 | ***Tatlockia micdadei (Legionella micdadei)*** | ***Tatlockia micdadei (Legionella micdadei)***  Herpes Simplex Virus 1 |
| 5 | ***Pseudomonas aeruginosa***  ***Stenotrophomonas maltophilia*** | ***Pseudomonas aeruginosa***  ***Stenotrophomonas maltophilia***  Human Herpesvirus 6 |
| 6 | ***Legionella longbeachae*** | ***Legionella longbeachae*** |
| 7 | ***Staphylococcus aureus*** | ***Staphylococcus aureus***  *Leuconostoc lactis*  *Rothia mucilaginosa* |
| 8 | ***Nocardia spp*** | ***Nocardia spp*** |
| 9 | ***Aspergillus spp***  ***Nocardia spp*** | ***Nocardia spp***  Prevotella melaninogenica  Human Adenovirus D |
| 10 | ***Pseudomonas aeruginosa*** | ***Pseudomonas aeruginosa*** |
| 11 | ***Serratia marcescens*** | ***Serratia marcescens*** |
| 12 | ***Staphylococcus aureus*** | ***Staphylococcus aureus*** |
| 13 | ***Staphylococcus aureus*** | ***Staphylococcus aureus*** |
| 14 | ***Aspergillus spp***  ***Stenotrophomonas maltophilia*** | ***Stenotrophomonas maltophilia***  *Rhizomucor pusillus* |
| 15 | ***Aspergillus spp***  *Achromobacter dentrificans*  *Achromobacter xylosoxidans* | ***Aspergillus spp***  *Aspergillus niger*  *Aspergillus tubingensis* |
| 16 | ***Aspergillus spp*** | ***Aspergillus spp***  *Aspergillus fumigatus*  BK Polyomavirus |
| 17 | **Mucor/Rhizomucor** | **Mucor/Rhizomucor**  *Rhizopus microsporus*  *Enterococcus faecalis*  *Lactobacillus rhamnosus*  *Rothia mucilaginosa*  *Staphylococcus epidermidis*  EBV |
| 18 | **Mucor/Rhizomucor** | **Mucor/Rhizomucor**  *Rhizomucor pusillus*  *Enterococcus faecalis* |
| 19 | ***Aspergillus fumigatus*** | ***Aspergillus fumigatus***  *Enterococcus faecium*  Human Herpesvirus 6 |

| 20 | ***Pneumocystis jirovecii*** | ***Pneumocystis jirovecii***  *Enterococcus faecium*  *Staphylococcus epidermidis*  Human Herpesvirus 7 |
| --- | --- | --- |
| 21 | **Mucor/Rhizomucor** | **Mucor/Rhizomucor**  *Rhizopus microsporus* |
| 22 | ***Rhizopus microsporus*** | ***Rhizopus microsporus*** |
| 23 | ***Pneumocystis jirovecii*** | ***Pneumocystis jirovecii*** |
| 24 | ***Pneumocystis jirovecii*** | ***Pneumocystis jirovecii*** |
| 25 | ***Pneumocystis jirovecii*** | ***Pneumocystis jirovecii*** |
| 26 | ***Pneumocystis jirovecii*** | ***Pneumocystis jirovecii***  Primate Erythroparvovirus 1  Herpes Simplex Virus 1  CMV |
| 27 | ***Aspergillus spp*** | ***Aspergillus spp***  *Aspergillus fumigatus*  *Rothia mucilaginosa*  *Staphylococcus hominis* |
| 28 | **Mucor/Rhizomucor** | **Mucor/Rhizomucor**  *Rhizomucor pusillus*  *Staphylococcus epidermidis* |
| 29 | ***Aspergillus spp*** | ***Aspergillus spp***  *Aspergillus calidoustus*  *Staphylococcus aureus*  *Staphylococcus epidermidis*  *Candida glabrata*  Primate Bocaparvovirus |
| 30 | **CMV** | **CMV**  *E. coli* |
| 31 | **Human Herpesvirus 6** | **Human Herpesvirus 6**  *Enterobacter cloacae*  *Rothia dentocariosa*  *Klebsiella pneumoniae* |
| 32 | **CMV** | **CMV**  Torque Teno Virus |
| 33 | Enterovirus/Rhinovirus | *Haemophilus Influenzae*  Herpes Simplex Virus 1 |
| 34 | *Aspergillus terreus* | *Rhizomucor Pusillus*  *Streptococcus mitis* |
| 35 | *Actinomyces spp,*  *Stenotrophomonas maltophilia* | *Bacteroides fragilis*  *Prevotella melaninogenica* |
| 36 | *Aspergillus spp* | *Bacteroides stercoris* |
| 37 | *Aspergillus fumigatus* | *Clostridium Perfringens*  *Enterocloster clostridioformis (Clostridium clostridioforme)*  *Enterococcus faecalis*  *Klebsiella pneumoniae* |
| 38 | *Scedosporium spp* | *Clostridium spp*  *E. coli*  *Staphylococcus epidermidis* |
| 39 | *Aspergillus spp* | CMV |
| 40 | *Aspergillus spp* | EBV |
| 41 | Enterovirus/Rhinovirus | *Enterobacter cloacae* |
| 42 | *Hemophilus parainfluenza* | Human Herpesvirus 6 |
| 43 | *Aspergillus terreus* | Human Herpesvirus 6  Pseudomonas aeruginosa |
| 44 | *Cryptococcus neoformans* | *Klebsiella pneumoniae* |
| 45 | *Aspergillus spp* | Merkel Cell Polyoma Virus |
| 46 | *Aspergillus fumigatus* | *Pantoea agglomerans* |
| 47 | Parainfluenza | *Propionibacterium acidifaciens*  *Streptococcus thermophilus*  *Veillonella parvula*  *Actinomyces spp*  *Fusobacterium nucleatum* |
| 48 | *Aspergillus spp* | *Pseudomonas aeruginosa* |
| 49 | SARS-CoV-2 | *Rothia dentocariosa*  *Rothia mucilaginosa* |
| 50 | *Aeromonas caviae* | *Staphylococcus epidermidis* |
| 51 | *Aspergillus spp* | *Staphylococcus epidermidis* |
| 52 | *Aspergillus spp* | *Staphylococcus haemolyticus* |
| 53 | *Actinomyces spp* | None |
| 54 | *Scopulariopsis brevicaulis* | None |
| 55 | *Aspergillus fumigatus* | None |
| 56 | *Aspergillus spp* | None |
| 57 | *Aspergillus spp* | None |
| 58 | *Aspergillus spp* | None |
| 59 | *Aspergillus spp* | None |
| 60 | *Aspergillus spp* | None |
| 61 | *Aspergillus spp* | None |
| 62 | *Aspergillus spp* | None |
| 63 | *Aspergillus spp* | None |
| 64 | *Aspergillus spp* | None |
| 65 | *Aspergillus spp*  *Staphylococcus aureus* | None |
| 66 | *Cryptococcus spp* | None |
| 67 | Enterovirus/Rhinovirus | None |
| 68 | Human Metapneumovirus | None |
| 69 | *Pseudomonas aeruginosa* | None |
| 70 | *Pseudomonas aeruginosa* | None |
| 71 | *Staphylococcus aureus* | None |
| 72 | *Staphylococcus aureus* | None |
| 73 | *Staphylococcus aureus* | None |
| 74 | None | *Acinetobacter spp*  *Streptococcus mitis* |
| 75 | None | *Klebsiella pneumoniae*  *Bacteroides fragilis*  *Bacteroides ovatus*  *Parrbacteroides distasonis* |
| 76 | None | *Legionella anisa*  BK Polyomavirus |
| 77 | None | *Legionella hackeliae*  EBV  Trichodysplasia spinulosa-associated Polyomavirus |
| 78 | None | *Nocardia cyriacigeorgica*  *Streptococcus thermophilus* |
| 79 | None | *Prevotella melaninogenica* |
| 80 | None | *Prevotella melaninogenica*  *Rothia mucilaginosa*  *Helicobacter pylori*  *Enterococcus faecalis* |
| 81 | None | *Prevotella melaninogenica*  *Streptococcus infantus* |
| 82 | None | *Pseudomonas aeruginosa* |
| 83 | None | *Pseudomonas aeruginosa* |
| 84 | None | *Pseudomonas aeruginosa*  EBV  *Bacillus cereus* |
| 85 | None | *Ralstonia picketii* |
| 86 | None | *Rothia spp* |
| 87 | None | *Rothia mucilaginosa*  *Streptococcus oralis*  *Corynebacterium amycolatum* |
| 88 | None | *Tatlockia micdadei (Legionella micdadei)*  BK Polyomavirus |
| 89 | None | *Aspergillus fumigatus*  *Corynebacterium jeikeium* |
| 90 | None | *Aspergillus fumigatus*  *Klebsiella pneumoniae*  *Rothia dentocariosa* |
| 91 | None | *Cunninghamella* |
| 92 | None | *Rhizomucor Pusillus* |
| 93 | None | *Pneumocystis jirovecii* |
| 94 | None | Pneumocystis jirovecii  *Enterococcus faecium*  EBV |
| 95 | None | *Pneumocystis jirovecii*  Human Mastadenovirus C |
| 96 | None | Human Herpesvirus 6  Hemolytic Staphylococcus  *Enterococcus faecium* |
| 97 | None | *Bacillus cereus*  *Bacillus thuringiensis* |
| 98 | None | *Bacteroides faecis*  *Bacteroides ovatus*  *Bacteroides thetaiotaomicron*  *E. coli* |
| 99 | None | *Bacteroides fragilis* |
| 100 | None | *Bacteroides fragilis*  *Campylobacter consisus*  *Campylobacter ureolyticus*  *Clostridium clostridioforme*  *Rothia mucilaginosa*  *Fusobacterium nucleatum* |

| 101 | None | *Bacteroides ovatus*  *Fusobacterium mortiferum* |
| --- | --- | --- |
| 102 | None | *Bacteroides vulgatus* |
| 103 | None | *Bacteroides vulgatus* |
| 104 | None | BK Polyomavirus |
| 105 | None | BK Polyomavirus |
| 106 | None | *Candida albicans* |
| 107 | None | CMV |
| 108 | None | CMV |
| 109 | None | CMV |
| 110 | None | CMV  *Bacteroides vulgatus*  *E. coli* |
| 111 | None | CMV  EBV |
| 112 | None | CMV  *Prevotella oris* |
| 113 | None | CMV  VZV |
| 114 | None | *E. coli* |
| 115 | None | *E. coli* |
| 116 | None | *E. coli* |
| 117 | None | *E. coli* |
| 118 | None | *E. coli* |
| 119 | None | *E. coli* |
| 120 | None | EBV |
| 121 | None | EBV |
| 122 | None | *Enterobacter cloacae*  *Staphylococcus epidermidis* |
| 123 | None | *Enterococcus faecium* |
| 124 | None | *Enterococcus faecium* |
| 125 | None | *Enterococcus faecium* |
| 126 | None | *Enterococcus faecium* |
| 127 | None | *Enterococcus faecium*  BK Polyomavirus  Human Herpesvirus 6 |
| 128 | None | *Enterococcus faecium*  *Candida glabrata*  *Staphylococcus epidermidis* |
| 129 | None | *Enterococcus faecium*  *Lactobacillus rhamnosus*  *Candida albicans*  Human Herpesvirus 6 |
| 130 | None | *Enterococcus faecium*  *Staphylococcus epidermidis*  *E. coli* |
| 131 | None | *Fusobacterium nucleatum* |
| 132 | None | Herpes Simplex Virus 1 |
| 133 | None | Human Herpesvirus 6  CMV |
| 134 | None | Human Herpesvirus 6  *Granulicatella adiacens*  *Rothia dentocariosa*  *Rothia mucilaginosa*  *Streptococcus mitis*  *Streptococcus parasanguinis* |
| 135 | None | Human Herpesvirus 6 |
| 136 | None | JC Polyomavirus  Torque Teno Virus |
| 137 | None | *Klebsiella pneumoniae* |
| 138 | None | *Lactobacillus fermentum* |
| 139 | None | *Lactobacillus rhamnosus* |
| 140 | None | *Lactobacillus rhamnosus*  *Rothia mucilaginosa* |
| 141 | None | *Lactobacillus spp*  *Rothia spp*  *Streptococcus Thermophilus*  *Veillonella Parvula*  *Saccharomyces spp* |
| 142 | None | *Parabacteroides johnsonii* |
| 143 | None | *Parvula*  *Prevotella melaninogenica*  *Streptomyces cattleya*  *Veillonella dispar* |
| 144 | None | *Pseudomonas aeruginosa*  Torque Teno Virus  Trichodysplasia spinulosa-associated Polyomavirus |
| 145 | None | *Rothia mucilaginosa* |
| 146 | None | *Rothia mucilaginosa* |
| 147 | None | *Serratia ureilytica* |
| 148 | None | *Staphylococcus aureus* |
| 149 | None | *Staphylococcus epidermidis* |
| 150 | None | *Streptococcus mitis* |
| 151 | None | *Streptococcus oralis* |
| 152 | None | *Veillonella parvula* |
| 153 | None | None |
| 154 | None | None |
| 155 | None | None |
| 156 | None | None |
| 157 | None | None |
| 158 | None | None |
| 159 | None | None |
| 160 | None | None |
| 161 | None | None |
| 162 | None | None |
| 163 | None | None |
| 164 | None | None |
| 165 | None | None |
| 166 | None | None |
| 167 | None | None |
| 168 | None | None |
| 169 | None | None |
| 170 | None | None |
| 171 | None | None |
| 172 | None | None |
| 173 | None | None |
| 174 | None | None |
| 175 | None | None |
| 176 | None | None |
| 177 | None | None |
| 178 | None | None |
| 179 | None | None |
| 180 | None | None |
| 181 | None | None |
| 182 | None | None |
| 183 | None | None |
| 184 | None | None |
| 185 | None | None |
| 186 | None | None |
| 187 | None | None |
| 188 | None | None |
| 189 | None | None |
| 190 | None | None |
| 191 | None | None |
| 192 | None | None |
| 193 | None | None |
| 194 | None | None |
| 195 | None | None |
| 196 | None | None |
| 197 | None | None |
| 198 | None | None |
| 199 | None | None |
| 200 | None | None |
| 201 | None | None |
| 202 | None | None |
| 203 | None | None |
| 204 | None | None |
| 205 | None | None |
| 206 | None | None |
| 207 | None | None |
| 208 | None | None |
| 209 | None | None |
| 210 | None | None |
| 211 | None | None |
| 212 | None | None |
| 213 | None | None |
| 214 | None | None |
| 215 | None | None |
| 216 | None | None |
| 217 | None | None |
| 218 | None | None |
| 219 | None | None |
| 220 | None | None |
| 221 | None | None |
| 222 | None | None |
|  | | |
